# Development and patterning of a highly versatile visual system in spiders

**DOI:** 10.1098/rspb.2024.2069

**Published:** 2025-03-12

**Authors:** Luis Baudouin Gonzalez, Anna Schönauer, Amber Harper, Saad Arif, Daniel J. Leite, Philip O. M. Steinhoff, Matthias Pechmann, Valeriia Telizhenko, Atal Pande, Zoe X. Schultz, Carolin Kosiol, Madeleine Aase-Remedios, Alistair P. McGregor, Lauren Sumner-Rooney

**Affiliations:** ^1^ Oxford University Museum of Natural History, University of Oxford, Parks Road, Oxford OX1 3PW, UK; ^2^ Department of Biological and Biomedical Sciences, Oxford Brookes University, Gipsy Lane, Oxford OX3 0BP, UK; ^3^ Enara Bio, Science Park, Bellhouse Building Level 3, Sanders Rd, Littlemore, Oxford OX4 4GA, UK; ^4^ Department of Biosciences, Durham University, Stockton Road, Durham DH1 3LE, UK; ^5^ Zoologisches Institut und Museum, Universität Greifswald, Loitzer Strasse 26, Greifswald 17489, Germany; ^6^ Department of Developmental Biology, Universität zu Köln, Zuelpicher Strasse 47B, Köln 50674, Germany; ^7^ School of Biology, St Andrews University, St Andrews KY16 9ST, UK; ^8^ Leibniz Institute for Biodiversity and Evolution, Museum für Naturkunde, Invalidenstrasse 43, Berlin 10115, Germany

**Keywords:** eye development, spiders, evolutionary neurobiology, visual systems, retinal determination

## Abstract

Visual systems provide a key interface between organisms and their surroundings, and have evolved in many forms to perform diverse functions across the animal kingdom. Spiders exhibit a range of visual abilities and ecologies, the diversity of which is underpinned by a highly versatile, modular visual system architecture. This typically includes eight eyes of two developmentally distinct types, but the number, size, location and function of the eyes can vary dramatically between lineages. Previous studies of visual system development in spiders have confirmed that many components of the retinal determination gene (RDG) network are conserved with other arthropods, but so far, comparative studies among spiders are lacking. We characterized visual system development in seven species of spiders representing a range of morphologies, visual ecologies and phylogenetic positions, to determine how these diverse configurations are formed, and how they might evolve. Combining transcriptomics, *in situ* hybridization, and selection analyses, we characterize the repertoires and expression of key RDGs in relation to adult morphology. We identify key molecular players, timepoints and developmental events that may contribute to adult diversity, in particular the molecular and developmental underpinnings of eye size, number, position and identity across spiders.

## 1. Background

Eyes have evolved many times in a wide variety of forms suited to their respective needs [1]. Visual system configuration is also highly variable: although many taxa have one pair of eyes, many insects have ocelli in addition to compound eyes [2], while molluscs may have hundreds of eyes [3,4]. Different configurations presumably offer different advantages, but their evolution remains understudied [5,6].

**Figure 1.**
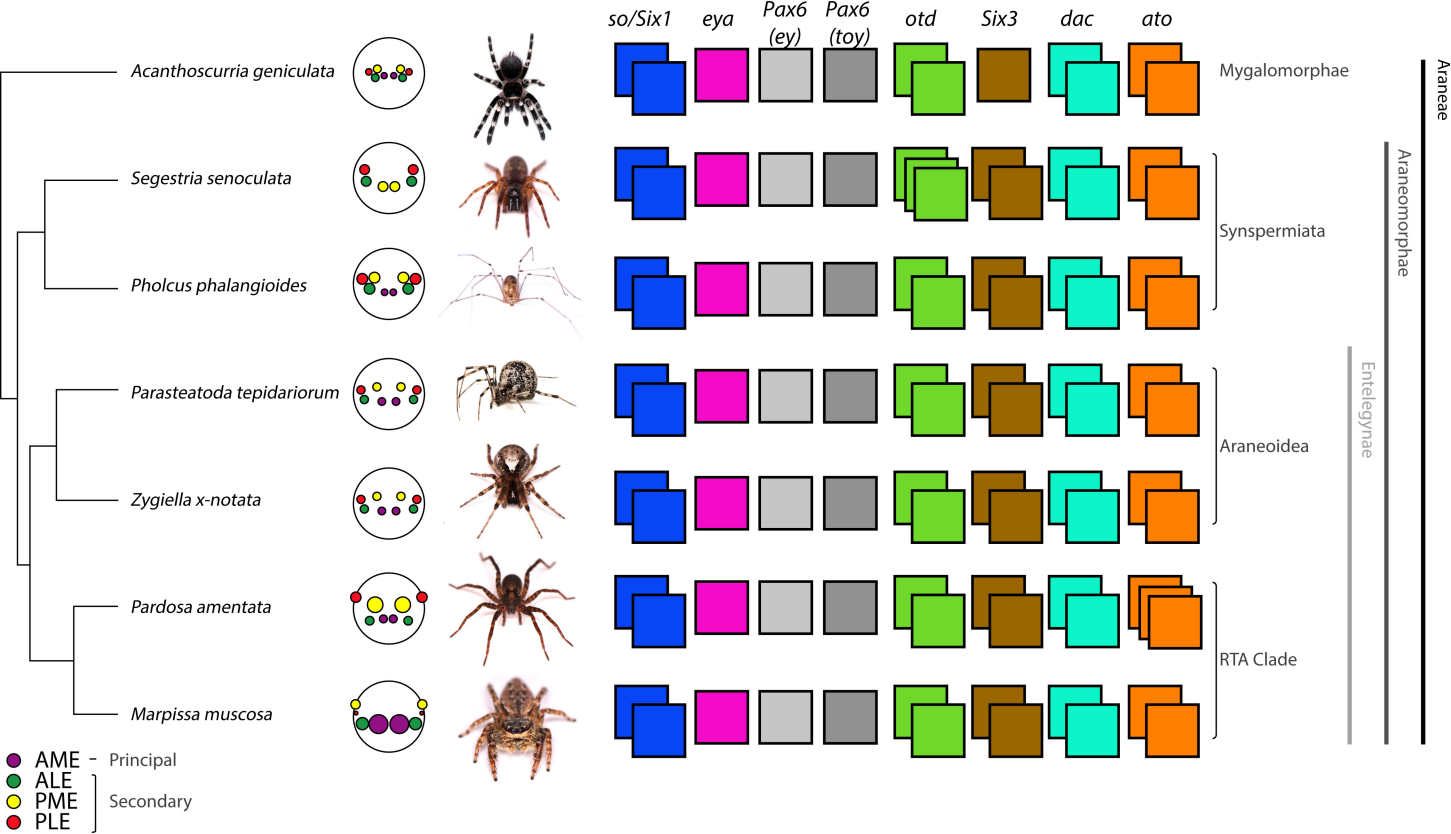
Retinal determination gene repertoires in spiders. RDG duplicates were highly conserved, except *eya*, which was always present in single-copy, and additional duplicates of otd in *Segestria senoculata* and ato in *Pardosa amentata*. Note that the posterior-most eye pair in *M. muscosa* is the PMEs. Images provided by Sam J. England, Grace Blakeley (*P. tepidariorum*) and Matthias Pechmann (*A. geniculata*). Topology and branch lengths from [9]. *Ato, atonal; dac, dachshund; ey, eyeless; eya, eyes absent; otd, orthodenticle; so, sine oculis; toy, twin of eyeless.*

Arthropods exhibit extraordinary diversity in both the structure and function of their visual systems, from loose clusters of single-aperture ocelli to highly sophisticated compound eyes [7]. Spiders provide an excellent opportunity to study how and why these architectures diversify. Most spiders have four pairs of eyes of two distinct types: one pair of ‘principal’ eyes, homologous to other arthropod median eyes, including insect ocelli; and three pairs of ‘secondary’ eyes, homologous to other arthropod lateral eyes including insect compound eyes [8]. The individual pairs are conventionally named after their position on the cephalothorax; the principal eyes being the anterior median eyes (AMEs) and the secondary eyes comprising the anterior lateral (ALEs), posterior lateral (PLEs) and posterior median eyes (PMEs), despite the latter being homologous to other arthropod lateral eyes. This provides the basis for a highly versatile modular system (figure 1) with substantial variation in eye size, number, position and function [8], facilitating diverse ecologies and behaviours [10]. Despite this diversity, spider visual systems remain united by a conserved blueprint spanning hundreds of millions of years of divergence [8].

Diversification in organ size, number and location may have direct functional and fitness impacts, and is underpinned by changes to developmental processes. The determination of these properties has occupied developmental biologists studying structures ranging from *Drosophila* genitalia to stickleback spines [11,12]. The same gene regulatory networks often control organ development, but changes in spatio-temporal gene expression affecting cell number and size, for example, may produce substantial changes to adult morphology [13].

Visual system development is regulated by highly conserved retinal determination gene (RDG) networks: transcription factors including PAX6, Orthodenticle (Otd), and Sine oculis (So) control eye development in vertebrates, insects and molluscs [14]. In insects, many core RDGs, including *Pax6*, *so*, *eyes absent* (*eya*) and *atonal* (*ato*), as well as the Wingless and Hedgehog signalling pathways, are employed in the larval eyes, ocelli and compound eyes [15]. However, the roles and interactions of RDGs vary between different visual organs within species and in homologous organs between species [15–17].

Compared to insects, we know relatively little about visual system development and how it has facilitated morphological and functional diversity in most spiders. Studies of *Parasteatoda tepidariorum* and *Cupiennius salei* [18–20] recovered many orthologues of the *Drosophila* RDG network [21,22], including duplicates likely resulting from an ancient whole-genome duplication (WGD) in arachnopulmonates [23]. These demonstrated RDG expression in the eye primordia, supported the homology of spider eyes with insect ocelli and compound eyes*,* and proposed a combinatorial RDG expression mechanism to determine the identity of the four eye pairs. However, there were also striking differences implying that RDG network structure is not necessarily conserved, even where orthologous genes are expressed [18–20].

These studies suggest several mechanisms for visual system diversification: distinct developmental origins of the two eye types, duplicated RDGs, eye-specific combinations of RDGs expressed and different spatiotemporal expressions of RDGs, could all contribute to divergence between taxa and eye pairs. However, this comparative approach requires broader phylogenetic data to identify correlations with morphological and functional variation. We characterized visual system development in seven species representing the major clades of extant spiders (Mygalomorphae, Synspermiata, Araneoidea, and the retrolateral tibial apophysis or RTA clade) and a range of morphologies and visual ecologies to identify developmental mechanisms underpinning four key characteristics: eye size, number, location and function (figure 1): *Acanthoscurria geniculata*, a tarantula with eight small eyes at the centre of the cephalothorax; *Segestria senoculata*, a tube-dwelling member of the early-diverging clade Synspermiata that lacks AMEs; *Pholcus phalangioides*, another synspermiatan that hunts in an aerial web and has eight eyes, with the secondary eyes grouped in two triads; *P. tepidariorum* and *Zygiella x-notata*, both orb weavers with eight eyes of roughly equal size encircling the anterior cephalothorax; *Marpissa muscosa*, a jumping spider with greatly enlarged AMEs, large ALEs and PMEs but vestigial PLEs; and *Pardosa amentata*, a wolf spider with enlarged posterior and small anterior eyes. Both *M. muscosa* and *P. amentata* belong to the RTA clade and are thought to use vision to hunt and court. In the remaining species, these behaviours are primarily mediated by mechano- and chemoreception; the function of the eyes is unknown but may include habitat selection, the detection of shadows, and the regulation of circadian rhythms [24,25].

## 2. Methods

### (a) Animal collection and culture

Adult *M. muscosa*, *P. amentata*, *P. phalangioides*, *S. senoculata* and *Z. x-notata* were collected from Berlin and Greifswald, Germany, and Oxford, UK, and kept at 25°C with a 12:12 h light:dark cycle. *P. tepidariorum* embryos were collected from an in-house culture at Oxford Brookes University under the same conditions, and *A. geniculata* embryos were collected from an in-house culture at the Universität zu Köln [26]. An egg sac of *D. cupreus* was collected from a drystone wall in West Yorkshire, UK.

### (b) RNA extraction and transcriptome assembly

Total RNA was extracted from mixed-stage embryos of *Z. x-notata*, *S. senoculata* and *D. cupreus* using QIAzol following standard protocol (Qiagen). Libraries were prepared using a TruSeq RNA kit (including polyA selection) and sequenced using NovaSeq (100 bp PE, Edinburgh Genomics). Erroneous k-mers and uncorrectable read pairs were removed using rCorrector v.1.0.4 (default settings) [27] and custom Python script (https://github.com/harvardinformatics/TranscriptomeAssemblyTools/blob/master/FilterUncorrectabledPEfastq.py, courtesy of Adam Freeman). Adapter sequences were removed, and low-quality ends trimmed using TrimGalore! v.0.6.5 (phred cutoff = 5) [28]. Quality was assessed using FastQC v.0.11.9 [29]. Processed reads were used for *de novo* transcriptome assembly with Trinity v. 2.11.0 (default settings) [30]. Completeness was assessed with Busco v. 5.0.0 [31] using longest isoforms (default settings, arachnida_obd10 lineage).

### (c) Identification and phylogenetic analysis of RDGs

RDGs were identified by tBLASTn [e-value 0.05, 31], using protein sequences from *P. tepidariorum* [19,20] as queries against the available transcriptomes of *A. geniculata* (PRJNA588224), *P. phalangioides* (Turetzek N, Torres-Oliva M, Kaufholz F, Prpic NM, Posnien N, 2017, unpublished data), *M. muscosa* (PRJNA707377), *P. amentata* (PRJNA707377), *Charinus acosta* (PRJNA707377) and *Euphrynichus bacillifer* (PRJNA707377), and the newly assembled transcriptomes of *Z. x-notata*, *S. senoculata* and *D. cupreus* Protein sequences were predicted using ORFfinder (https://www.ncbi.nlm.nih.gov/orffinder/) using ‘any-sense codon’ to retrieve sequences from fragmented transcripts.

To confirm RDG identity and orthology, we performed phylogenetic analysis using full-length protein sequences from 12 spiders (*A. geniculata, Araneus ventricosus, Argiope bruennichi, C. salei, D. cupreus, M. muscosa, P. amentata, P. phalangioides, P. tepidariorum, S. senoculata, Stegodyphus dumicula, Z. x-notata*), two amblypygids (*C. acosta, E. bacillifer*), a scorpion (*Centruroides sculpturatus*), a tick (*Ixodes scapularis*) and two insects (*D. melanogaster, Tribolium castaneum*) (electronic supplementary material, files 1–6, table S2). Sequences from *P. tepidariorum* and *C. salei* were retrieved from previous studies [18–20]. Sequences from *A. ventricosus, A. bruennichi, S. dumicula, C. sculpturatus, I. scapularis, T. castaneum* and *D. melanogaster* were retrieved from NCBI proteomes. Alignments used Clustal Omega (default settings, https://www.ebi.ac.uk/Tools/msa/clustalo/). Phylogenies were generated using RAxML-NG v.1.0.2 [32], with ModelTest-NG v.0.1.7 for model selection (*ato:* JTT+I+G4+F, *otd*: JTT+I+G4+F, Six: JTT+I+G4+F, *dac*: PMB+I+G4+F, *eya*: JTT+I+G4+F) and automatic bootstrapping. To achieve sufficient resolution of Pax phylogeny, we inferred a maximum-likelihood phylogeny of Pax4/6/10-ey/toy/eyg/toe and Pax3/7-prd/gsb/gsbn full amino acid sequences from a larger bilaterian dataset from previous analyses [18,33], updated to include all genes from each species and reflect current availability of resources on GenBank (electronic supplementary material, tables S3 and S4). Sequences were aligned with MUSCLE and the alignment was manually trimmed to include both the paired domain and homeodomain, as well as conserved regions outside these, while removing low-occupancy positions in the alignment (electronic supplementary material, S4), before running IQ-Tree with ModelFinder and 1000 ultrafast bootstrap replicates [34–36].

### (d) Cloning and probe synthesis

cDNA synthesis used the QuantiTect reverse transcription kit (Qiagen). Gene fragments were amplified by PCR and cloned into pCR^®^4-TOPO^®^TA vectors (ThermoFisher Scientific). See electronic supplementary material, table S1 for primers. RNA probes were synthesized using T7 (10881775001, Roche) or T3 RNA polymerase (11031163001, Roche), depending on fragment orientation, using DIG RNA labelling mix (11277073910, Roche).

### (e) *In situ* hybridization (ISH)

Embryos were staged after Mittmann & Wolff [37] and fixed for *in situ* hybridization (ISH) following Akiyama-Oda *et al*. [38], with minor modifications [39], at stages 9.1/9.2, 10, 11, 12, 13.1 and 13.2, when available. Whole-mount ISH followed Prpic *et al*. [40] with minor modifications [39]. Embryos were counterstained with DAPI (1:2000; 10236276001, Roche) for approximately 20 min and stored in PBS-T at 4°C. Imaging used Zeiss Axio Zoom V.16 and Photoshop CS6 (Adobe).

### (f) Selection on RDG sequences

We queried genes and transcripts from the above species against four additional spider genomes: *Dysdera silvatica* (GCA_006491805.2)*, Ectatosticta davidi* [41], *Latrodectus elegans* (GCA_030067965.1) and *Oedothorax gibbosus* (GCA_019343175.1). We used Blastn [42] to identify candidate sequences (e-value 0.05), which were extracted with samtools [43] (transcriptomes and genome annotations) or bedtools getfasta [44](genomes). Hits from genome annotations were collapsed by gene ID, whereas hits from transcriptomes and genomes were collapsed using cd-hit-lap [45] and cluster threshold 90%. Where multiple hits were recovered, non-overlapping fragments were eliminated, keeping the longest fragments overlapping the most conserved regions.

Sequences were translated into amino acids and aligned using L-INS-i in MAFFT 7 [46] to preserve the reading frame. Corresponding alignments of nucleotide coding sequences were built using PAL2NAL [47]. Regions that were predominantly gaps or poorly aligned were removed with Gblocks [48]. Phylogenies were constructed using IQ-TREE [35] with automatic model selection [36] and ultrafast bootstrapping [49] (electronic supplementary material, figures S1–S6).

We used CodeML in PAML 4 [50] to characterize selection on coding sequences, comparing nonsynonymous : synonymous substitutions (dN/dS or ω) for different scenarios. First, we used the branch model [51,52], assuming different ω ratio parameters for different lineages to evaluate selection on different sets of gene copies. Paralogous groups were labelled as foregrounds and tested sequentially for each gene. Significance was assessed using likelihood ratio tests (LRT) with null model M0 (uniform ω). Next, we performed branch tests on selected lineages with additional gene duplications or distinctive expression patterns. Finally, we applied the branch-site model to detect potential amino acid sites under positive selection using the Bayes empirical Bayes method [53]. Statistical significance was evaluated using LRT and twice the log-likelihood difference between null and alternative models, compared to a 50:50 mixture of a chi-square distribution (1 df) and a point-mass at 0 [54].

## 3. Results

### (a) Gross development of the eyes and CNS

The principal eye primordia (PEP) originate in the non-neurogenic ectoderm at the anterior tip of the head lobes and migrate posteriorly and ventrally during head closure [55]. The secondary eye primordia (SEP) appear at the ventro-lateral rim of the head lobes and divide into three between stages 10.2−12. These form epithelial indentations (visible from stage 13.1 in most species; electronic supplementary material, figure S7) that invaginate and become covered by the developing lens [55]. The secondary eye pairs are named by developmental homology: the dorsal-most pair migrate medially during head closure to form the PMEs, the ventral-median pair forms the ALEs, and the lateral pair forms the PLEs [19]. The developing CNS is visible in synchrotron scans from stage 12−13 (see electronic supplementary material, figure S7). By stage 13.1, the circumoesophageal ring is visible except in *A. geniculata* (electronic supplementary material, figure S7). Two prominent nerve bundles project anteriorly from the protocerebrum, which may contribute to the developing optic neuropils. However, connections to the eye primordia were not visible.

### (b) RDG repertoires are highly conserved

We identified orthologues of *so*, *Pax6*, *Otd*, *Six3*, *dac*, *eya* and *ato* (electronic supplementary material, table S2). As in *P. tepidariorum* and *C. salei* [18,19], we identified two copies in most species, except *eya* (figure 1). With the exception of *ato* and *Pax6*, gene tree topologies place duplications since the divergence of arachnopulmonates (electronic supplementary material, figures S1–S5). Given the arachnopulmonate WGD and the resolution of spider phylogeny within gene clades, we interpret these copies as ohnologues. Orthologues of *Pax6.1* belonged to the *eyeless* family, and orthologues of *Pax6.2* belonged to the *twin of eyeless* family (in line with [18]). *Pardosa amentata* expressed an additional copy of *ato; Pa-ato2.1* and *Pa-ato2.2* exhibited high nucleotide sequence similarity, suggesting very recent, lineage-specific duplication (electronic supplementary material, S1 and figure S1). We also identified a third copy of *otd* in *S. senoculata*; both *Ss-otd1.1* and *Ss-otd1.2* are orthologous to *Pt-otd1* (figure 1; electronic supplementary material, S3, figure S3). In *P. phalangioides*, here the closest relative of *S. senoculata*, both copies of *otd* are apparently orthologous to *Pt-otd1* (electronic supplementary material, figure S3), implying *otd1* duplication in Synspermiata and the loss of *otd2* in *P. phalangioides*. Only one copy of *Pax6*, orthologous to *Pt-Pax6.1* (and *eyeless*), was recovered in *D. cupreus* (electronic supplementary material, S4, figure S4), and one copy of *Six3*, orthologous to *Pt-Six3.2*, in *A. geniculata* (electronic supplementary material, S5, figure S5). Whether their paralogues are truly absent, or undetected due to the timing and level of expression or the depth, assembly and analysis of the transcriptomes cannot be confirmed.

### (c) Early expression of RDGs in P. tepidariorum

**Figure 2.**
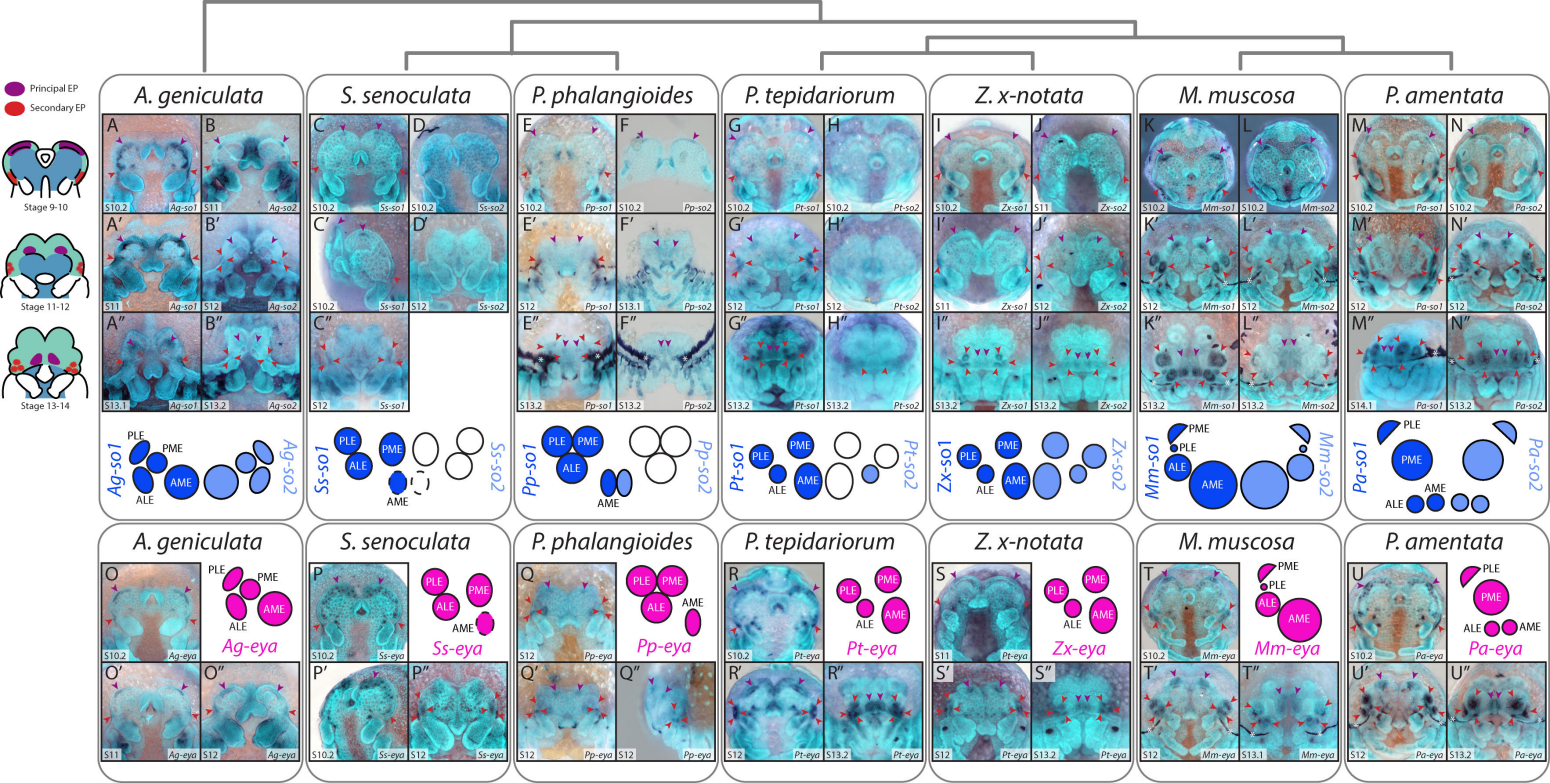
Expression of *sine oculis* and eya orthologues in developing spider embryos. orthologues of *so1* and *eya* were expressed in all developing eyes. *so2* expression was detected in all eyes in *Acanthoscurria geniculata, Zygiella x-notata, Marpissa muscosa* and *Pardosa amentata* and in the AMEs of *Pholcus phalangioides*. Purple arrowheads and schematics indicate PEP, red arrowheads and schematics indicate SEP and asterisks indicate artifactual staining of the developing cuticle (see electronic supplementary material, figure S10).

Leite *et al*. [56] described the expression of *Pt-Pax6.1* and *Pt-Pax6.2* at the anterior rim of the germ band from stage 6. To establish whether these could initiate eye development, we examined the earliest expression of the early RDGs, *Pt-so1*, *Pt-eya*, *Pt-otd2* and *Pt-Pax2.1* [19,20,56,57]. *Pt-eya* was expressed around the dorsal periphery from stage 8 and in the eye primordia by stage 10.2 (electronic supplementary material, figure S8A–S8B). Faint *Pt-so1* expression lined the perimeter of the head lobes at stage 8.2 (electronic supplementary material, figure S8C–S8D), then split, corresponding to the eye primordia by stage 10.1 (figure 2). *Pt-otd2* was expressed in the pre-cheliceral segment at stage 8.2 and only appeared in the PEP from stage 10.1 (electronic supplementary material, figure S8E–S8F). dFISH revealed brief overlap of *Pt-Pax6.2* and *Pt-otd2* expression in the developing brain, but not the PEP (electronic supplementary material, figure S8G–S8I). *Pt-Pax2.1* was expressed in the SEP from stage 10.1 (electronic supplementary material, figure S8J–S8L).

### (d) Expression of Pax6 orthologues

Previous work on *P. tepidariorum* detected *Pax6* expression in the developing brain but not the eye primordia (electronic supplementary material, figure S9) [19]. This was generally consistent across our study species (electronic supplementary material, figure S9A–S9L′), but both copies of *Pax6* were faintly expressed along the edge of the developing head in *M. muscosa* and *P. amentata*, as was *Ag-Pax6.2* in *A. geniculata* (electronic supplementary material, figures S9I–S9J′, S9B-B′). This *Ag-Pax6.2* expression could overlap with the eye primordia at stages 10.2−11, but not stage 10.1 (electronic supplementary material, figure S9B–S9B′). *Mm-Pax6.1* and *Pa-Pax6.1* expression was restricted to the non-neurogenic ectoderm between and proximal to the PEP and SEP at stage 10.2 (electronic supplementary material, figure S9I–S9I′). At stages 12−13.1, *Pa-Pax6.1* expression partially surrounded the AMEs, ALEs and PMEs (electronic supplementary material, figure S9K′–S9K″). *Mm-Pax6.2* and *Pa-Pax6.2* had similar expression patterns, with gaps corresponding to the SEP (electronic supplementary material, figures S9J–S9J′, S9L). *Mm-Pax6.2* expression in the non-neurogenic ectoderm may partially overlap with the PEP during stages 10.2−11 (electronic supplementary material, figure S9J–S9J′).

### (e) Expression of *sine oculis* orthologues

*Pt-so1* and *Pt-so2* expression patterns were consistent with [19]. From stage 10.2, a paired *Pt-so1* expression domain at the anterior edge of the head corresponds to the PEP, which migrate towards the final position of the AMEs by stage 13.2 (figure 2G–G″, purple arrowheads). A second pair of expression domains appears at stage 10.2 at the lateral edge of the head; these correspond to the SEP, which split into three at stage 12 to form the ALEs, PMEs and PLEs (figure 2G–G″, red arrowheads). *Pt-so2* expression was only detected in the ALEs (figure 2H–H″). orthologues of *Pt-so1* were expressed in all eye primordia of all species studied (figure 2), but the expression of *Pt-so2* orthologues was less consistent.

*Ag-so1* expression extended beyond the putative developing eyes, and expression in the SEP did not split into distinct domains by stage 13.1 (figure 2A–A′). *Ag-so2* expression was more restricted, and expression in the SEP split into three domains from stage 12 (figure 2B–B′).

In *P. phalangioides* and *S. senoculata*, expression domains of *Pp-so1*, *Pp-so2* and *Ss-so1* in the developing AMEs were smaller than orthologues in other species (figure 2C–F″). Intriguingly, *Ss-so1* expression was clearly visible in the region of the PEP at stage 10.2, despite the absence of AMEs in this family, but expression was no longer detected by stage 12 (figure 2C–C″). *Pp-so2* was only expressed in the PEP (figure 2F–F″) and *Ss-so2* was not expressed in any eye primordia (figure 2D–D′). In the remaining (entelgyne) species, orthologues of both *Pt-so1* and *Pt-so2* were detected in all eye primordia. *Zx-so1* expression was identical to *Pt-so1* (figures 2G–G″ and 4I–I″); its early expression domain was larger and stronger than *Zx-so2*, but became restricted to the edges of the secondary eyes during stage 13.

In *M. muscosa* and *P. amentata*, expression domains of both copies of *so* reflected adult eye sizes as early as stage 10: in *M. muscosa* expression in the developing AMEs was larger, and those in the lateralmost secondary eye were smaller (figure 2K–L″) than in other species. This indicates that the smallest salticid eye pair is homologous to the PLEs of other spiders, and not the PMEs as they have historically been described. The eyes previously described as the salticid PLEs are homologous to the PMEs in other species. We use this new nomenclature hereafter. Likewise, expression domains of *Pa-so1* and *Pa-so2* were larger in the posterior than the anterior eyes from stage 12 (figure 2M–N″). In *M. muscosa*, *Mm-so1* was more strongly expressed in a ventral region of the AMEs at stage 13.2 (figure 2K″), while *Mm-so2* expression was more uniform (figure 2L″). In the secondary eyes, expression of *Mm-so1* was strongest around the eye perimeter, with an additional dot at the centre of the ALEs and PMEs (figure 2K′–K″). *Mm-so2* expression in the ALEs and PMEs was restricted to the lateral edges of the eyes (figure 2L′–L″). In *P. amentata*, *Pa-so2* expression in the AMEs was more intense and possibly broader than *Pa-so1* expression (figure 2M–N′). *Pa-so1* expression was pronounced at the periphery and ventral centre of the PMEs and PLEs, while *Pa-so2* expression formed a less pronounced crescent and a central/ventral dot (figure 2M′–N″).

### (f) Expression of eyes absent orthologues

*Pt-eya* was expressed in all eye primordia from stage 10 [19] (figure 2R–R″). In *A. geniculata* and *S. senoculata*, *eya* expression in the SEP did not split into discrete domains at stage 12 (figure 2O–O″,P–P″). At stage 10.2, *Ss-eya* was expressed in a large region along the anterior edge of the head encompassing the PEP, as well as laterally around the SEP, but by stage 12, expression was only visible in the latter (figure 2P–P″). In the remaining species, expression of *eya* split into distinct SEP by stage 12 (figure 2Q–U″). Compared to other species, *Mm-eya* had larger expression domains in the AMEs, ALEs and PMEs (figure 2T–T″), with concentrated dots of expression within the ALEs and PMEs, and *Pa-eya* had larger expression domains in the PLEs and PMEs (figure 2U–U″). Expression domains of *Mm-eya* in the PLEs (figure 2T′–T″), and of *Pa-eya* in the AMEs and ALEs, were smaller (figure 2U′–U″).

### (g) Expression of orthodenticle orthologues

**Figure 3.**
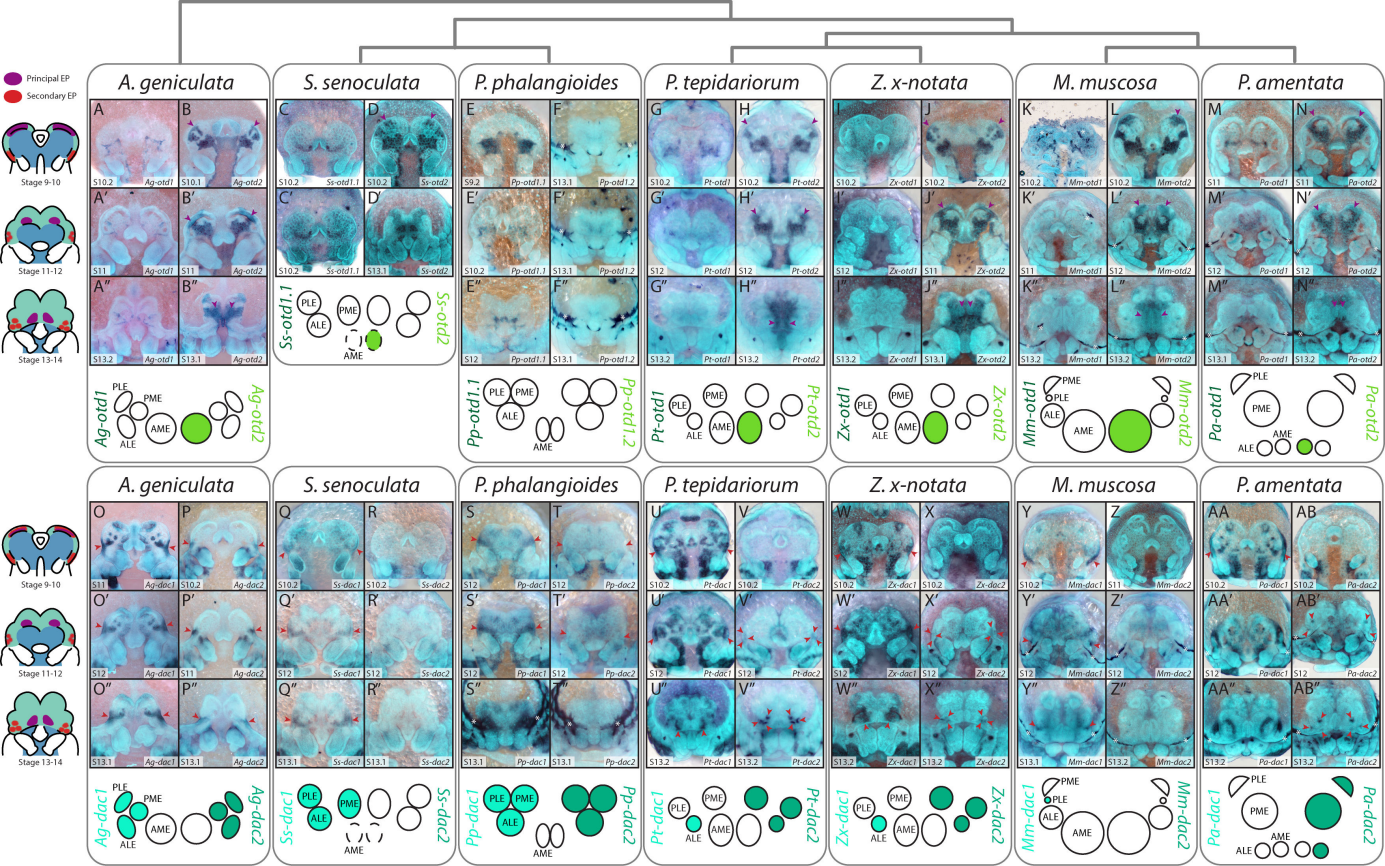
Expression of otd and *dac* orthologues in developing spider embryos. Two copies of *otd* were recovered, except in *Segestria senoculata*, which yielded three copies. *otd1* orthologues were not expressed in any developing eyes (including *Ss-otd1.2*, not pictured). *otd2* expression was detected from stage 10.2−11 in the AMEs of all species except *Pholcus phalangioides*. orthologues of *dac1* were expressed in all secondary eyes in *Acanthoscurria geniculata, S. senoculata* and *P. phalangioides*, but only in the ALEs of *Parasteatoda tepidariorum* and *Zygiella x-notata* and in the PLEs of *Marpissa muscosa*. Expression of *dac2* was detected in all SEP from stage 10.2−12, except in *M. muscosa* and *A. geniculata*. Purple arrowheads indicate PEP, red arrowheads indicate SEP and asterisks indicate artifactual staining of the developing cuticle.

In *P. tepidariorum*, *Pt-otd1* was not expressed in the eye primordia but in the developing brain at stage 10.2, becoming obscured during head closure (figure 3G–G″) [19]. *Pt-otd2* was expressed in the PEP and putative neural tissue from stage 10.2, becoming restricted to the midline during head closure (figure 3H–H″) [19]. These patterns were largely conserved across species. In *P. phalangioides*, *Pp-otd1.2* expression was faintly visible at the margin of the PEP (figure 3F″), but the depth of staining suggests this is within the developing brain, consistent with its orthology to *Pt-otd1* (electronic supplementary material, figure S3). *Ss-otd2* expression was detected in the region of the PEP at stage 10.2 (figure 3D), but was later restricted to the developing brain (figure 3D′). *Ss-otd1.2* produced no clear staining in any embryonic tissue and is not depicted. Expression of *Mm-otd2* in the PEP was distinctly larger and more intense compared to orthologues in other species from stage 10.2 (figure 3L′–L″ ).

### (h) Expression of *dachshund* orthologues

In *P. tepidariorum*, *dac* expression was restricted to the SEP. As described by [19], *Pt-dac1* was first expressed in the early (stage 10.2) SEP and later restricted to the ALEs (figure 3U–U″), while *Pt-dac2* was expressed in all SEP from stage 12 (figure 3V–V″).

The early expression of *Pt-dac1* in the SEP is apparently conserved (figure 3O,Q,S,U,W,Y,AB). However, differences emerged later, with only *Zx-dac1* showing identical expression to *Pt-dac1* (figure 3W–W″). *Ag-dac1*, *Ss-dac1* and *Pp-dac1* expression in the SEP persisted as one contiguous domain even after the splitting of *so* expression (figure 3O–O″, Q–Q″,S–S″). In *P. amentata*, *Pa-dac1* expression appeared to partially surround the PLEs and PMEs at stage 12 (figure 3AA′–AA″), and could partially overlap the primordia. *Mm-dac1* expression was restricted to the PLEs from stage 12 (figure 3Y–Y″).

Expression of *Pt-dac2* orthologues was more variable. *Ag-dac2* expression was visible in the SEP from stage 10.2; like *Ag-dac1*, it did not split into distinct domains (figure 3O–O″), but it was more restricted than *Ag-dac1* expression (figure 3O–P″). Likewise, *Pp-dac2* was expressed in the SEP from stage 12, but did not divide into distinct domains (figure 3T–T″). *Zx-dac2* expression was identical to *Pt-dac2* (figure 3X–X″). *Pa-dac2* expression was detected in all secondary eyes but did not reflect the size of the eyes as seen for *so* and *Six3.2* (figure 3AB–AB′). *Ss-dac2* and *Mm-dac2* expression were absent from all eye primordia (figure 3R–R″,Z–Z″).

### (i) Expression of Six3 orthologues

**Figure 4.**
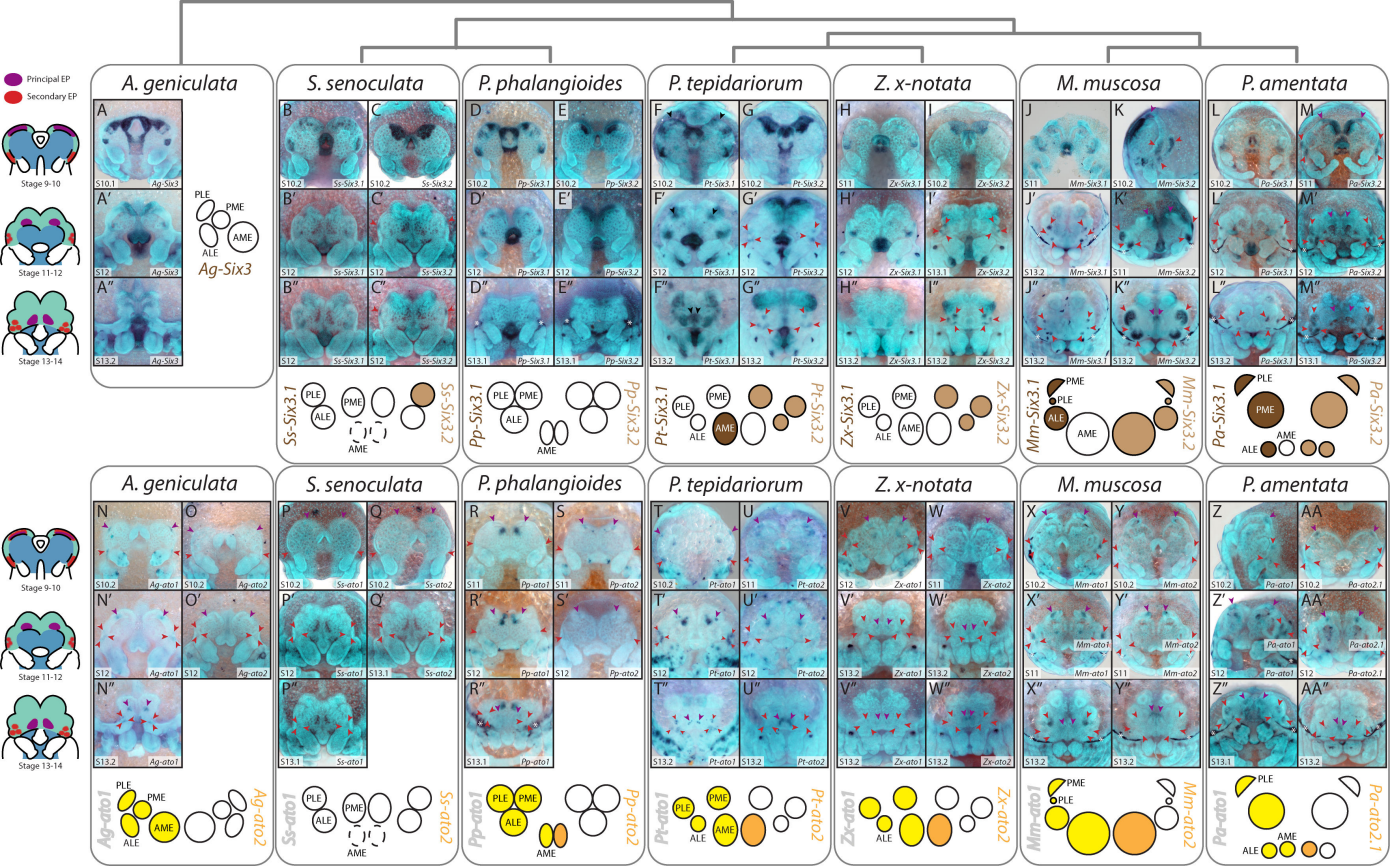
Expression of *Six3* and *ato* orthologues in developing spider embryos. *Six3.1* orthologues were expressed in the secondary eyes of RTA clade species and the AMEs of *Parasteatoda tepidariorum*. Expression of *Six3.2* orthologues was detected in all SEP, from stage 10.2−12, in entelegynes, plus the PLEs of *Segestria senoculata* and the AMEs of the RTA clade. *ato1* orthologues were expressed in all eye primordia from stage 10.2, except in *S. senoculata*. Expression of *ato2* was restricted to the PEP, and was absent in *S. senoculata* and *Acanthoscurria geniculata. Purple* arrowheads indicate PEP, red arrowheads indicate SEP and asterisks indicate artifactual staining of the developing cuticle.

Previous descriptions reported no *Pt-Six3.1* expression in the eye primordia of *P. tepidariorum*, while *Pt-Six3.2* expression was detected in all SEP from stage 12 (figure 4F–G′) [19]. We observed two small expression domains of *Pt-Six3.1* at the edge of the non-neurogenic ectoderm from stage 10.2 and subsequently more posteriorly as the non-neurogenic ectoderm grows over the neurogenic ectoderm (figure 4F–F″). At stage 12 it was reminiscent of *Pt-so1* expression in the AMEs.

*Six3* orthologue expression was somewhat conserved between species, but the expression of these genes was the most variable we studied.

In *A. geniculata* and *P. phalangioides* no *Six3* expression was detected in any eye primordia (figure 4A–A′,D–E′). In *S. senoculata*, *Ss-Six3.1* was not expressed in any eye primordia, but *Ss-Six3.2* expression may overlap with the developing PLEs at stage 12 (figure 4C′–C″). *Zx-Six3.2* was expressed in all SEP by stage 13.1, but *Zx-Six3.1* expression was not visible in any (figure 4H–I″). In *M. muscosa* and *P. amentata*, both *Six3* copies were detected in all SEP, but their spatiotemporal expression differed (figure 4J–M″): *Mm-Six3.1* and *Pa-Six3.1* expression was restricted to a small region at the centre of each eye and only visible from stage 12 onwards (figure 4J–J″,L–L″). Three distinct domains of *Mm-Six3.2* and *Pa-Six3.2* expression were already visible at stage 10.2/11 (figure 4K–K′,M). By stage 13.2, the domain of *Mm-Six3.2* in the ALEs and PMEs was larger and more intense than in the PLEs (figure 4K”), and *Pa-Six3.2* expression domains were larger in the PMEs and PLEs than the ALEs (figure 4M″). The larger expression domains of *Mm-Six3.2* surrounded *Mm-Six3.1* expression in the PMEs and ALEs (figure 4J″,K″). *Mm-Six3.2* and *Pa-Six3.2* were also expressed in the AMEs, but were restricted to a small dorsal area in *M. muscosa* (figure 4K–K″,M–M″).

### (j) Expression of atonal orthologues

*Pt-ato1* was expressed within a few cells in all developing eyes from stage 10.2 (figure 4T–T″) [20]. Although we did not previously detect *Pt-ato2* expression in the eyes, here we observed restricted expression of *Pt-ato2* within the PEP (figure 4U–U″).

This pattern was generally conserved, with *Pt-ato1* orthologues expressed in all eye primordia and *Pt-ato2* orthologues expressed only in the PEP (figure 4). However, we did not detect any expression of *Ag-ato2* in the eye primordia in *A. geniculata* (figure 4O–O′) or either copy of *ato* in *S. senoculata* (figure 4P–Q′). In *P. phalangioides*, expression of *Pp-ato1* in the SEP did not split into distinct domains at stage 12, and expression was no longer detected by stage 13.1 (figure 4R–R″). *Zx-ato1* and *Zx-ato2* expression (figure 4V–W″) was similar to *P. tepidariorum*. In *M. muscosa*, we did not detect expression of *Mm-ato1* in the PLEs (figure 4X–X″). Expression domains of *Mm-ato1* and *Pa-ato1* (figure 4Z–Z″) were larger than *Pt-ato1*.

### (k) Selection on RDG sequences

All genes were highly conservative and no instances of positive selection (ω > 1) were identified; however, there were statistically significant differences in ω for *ato* and *otd* duplications. *ato2* (ω = 0.066) and *otd1* (ω = 0.053) exhibited significantly higher rates of non-synonymous change than *ato1* (ω = 0.039) and *otd2* (ω = 0.017), respectively.

Next, we evaluated ω for *ato* paralogues in *P. amentata* and *otd* in *S. senoculata* were tested due to additional duplications, and for *Mm-dac2, Six3* in *M. muscosa* and *P. amentata*, and *so2* due to their expression patterns. We also tested *otd* in *P. phalangioides* and *Pax6* in *A. geniculata*, where ohnologues are lost. Significant increases in ω were detected for *Mm-dac2* (ω1 = 0.07, ω0 = 0.028), *Ag-Pax6* (ω1 = 0.08, ω0 = 0.019), *Ag-so2* (ω1 = 0.09, ω0 = 0.03) and *Pp-so2* (ω1 = 0.124, ω0 = 0.03). No additional synonymous substitutions were detected in *Ss-so2* compared to its nearest neighbour, *Pp-so2*; an exact value of ω is therefore difficult to estimate, but this branch is likely subject to positive selection. Significant decreases in ω, indicating lower selective pressure, were detected in *Ss-otd2* (ω1 = 0.011, ω0 = 0.036), *Pa-Six3.2* (ω1 = 0.0001, ω0 = 0.016), *Zx-so2* (ω1 = 0.006, ω0 = 0.031) and *Pt-so2* (ω1 = 0.01, ω0 = 0.031). See electronic supplementary material, table S4.

Positively selected codons were identified in *Mm-dac2* at sites 473 (glycine to glutamine) and 502 (glutamine to lysine)*,* and *Ag-so2* at sites 7 (alanine to asparagine), 209 (histidine to alanine) and 226 (glutamine to phenylalanine). See electronic supplementary material, table S5.

## 4. Discussion

These data provide much-needed insight to the genetic origins of visual system variation in spiders. Despite strong conservation in RDG repertoires and copy numbers, we report substantial differences in spatiotemporal gene expression that could underpin key aspects of morphological, functional and— ultimately—ecological diversity, in line with previous studies of organ size, number and placement. Our data also suggest that RDGN structure can vary, even when orthologous genes are involved, as the suites of expressed genes were not always consistent across eyes and species.

### (a) Eye number

Although most spiders have eight eyes, pairs can be lost at varying phylogenetic depths, from large clades to individual cavernicolous species [58]. This most commonly affects the AMEs, presumably owing to their distinct developmental origins and regulatory networks, and seems to occur frequently in Synspermiata. Despite the loss of the AMEs seen in *S. senoculata*’s probably occurring at the base of Dysderoidea around 100−150 Ma [9], we detected expression of *otd*, *eya* and *so* in the PEP. This is reminiscent of eyeless subterranean species such as *Astyanax mexicanus*, wherein the eyes begin developing before their eventual degradation (see [59–61] for reviews). However, such losses are generally more recent, across only hundreds of thousands of years [62]. Persistent RDG expression in *S. senoculata* PEP suggests pleiotropy in these genes or upstream factors [62]. Gainett *et al*. [63] recently demonstrated a similar phenomenon in the harvestman, where RDG and opsin expression revealed vestigial eyes from an even older loss. The *otd1* duplication in *P. phalangioides* and *S. senoculata*, both synspermiatans, offers a tempting lead: *otd1* is subject to greater positive selection than *otd2* across all spiders, and the *otd1* duplicates in *P. phalangioides* and *S. senoculata* exhibited relatively long branches (electronic supplementary material, figure S3). Notably, this is not the paralogue expressed in the AMEs; the *otd2* paralogue is absent from *P. phalangioides* and still expressed in *S. senoculata*. The high levels of pleiotropy in many RDGs, including *otd*, preclude further speculation or interpretation at this stage. Nevertheless, the correlation of this duplication with AME instability in Synspermiata warrants investigation of *otd* function in eight- and six-eyed taxa. Tandem duplication and retention of *otd1* could reduce stabilising selection on *otd2*; indeed, *Ss-otd2* exhibited long branches and relaxed selection despite its expression in the early PEP (electronic supplementary material, table S3, figure S3).

Where secondary eyes are lost, the most parsimonious explanation is failure of the primordia to split into three distinct eye fields. Microstructural examination of *Tetrablemma*, for example, reveals eyes merged beneath shared lenses (L.S.-R. 2020, unpublished observation).

### (b) Eye size

Eye size is among the most striking sources of variation in spider visual systems, has direct functional implications for contrast sensitivity and achievable spatial resolution, and correlates to ecology [64]. A recent comparative study demonstrated negative static allometry in eight different families [64], indicating that eye size is established early in life. Indeed, in salticids, eye (lens) diameter is already established in hatchlings and exhibits negative ontogenetic allometry, but photoreceptor numbers remain stable [65]. Our results demonstrate that differences in eye size are determined early in development: in *M. muscosa*, the large AMEs are apparent from *Mm-otd2* and *Mm-so1* expression by stage 10.2. This could result from the activation of RDG expression in more cells, from increased cell proliferation within these regions, or both. Both *M. muscosa* and *P. amentata* exhibit enlargement in, and size variation between, the secondary eye pairs. This enlargement is visible in the size of the SEP prior to division, particularly from *Mm-so2, Pa-so2* and *so2* (as *six1a*) in *C. salei*, another visual hunter within the RTA clade [17: figure 2*e*]. The division of the SEP is also distinctly uneven, meaning the interocular variation is established immediately and reducing the need for unequal rates of cell proliferation in the resultant eye fields. Control of field splitting remains unknown. In vertebrates, *hedgehog (hh*) contributes to the division of the optic vesicle by suppression between the eyes [66]; while Baudouin-Gonzalez *et al*. [20] detected no *hh* expression within the SEP of *P. tepidariorum*, a second copy of *hh* was recently detected in the SEP of *P. tepidariorum, P. phalangioides, P. amentata* and the mygalomorph *Ischnothele caudata* [67], and in the PEP of *P. amentata*, *P. phalangioides* and *I. caudata*. In *P. amentata*, *hh2* expression reflects size differences in the secondary eyes at stage 12, as observed for *Pa-so1*, *Pa-eya* and *Pa-Six3.2*. Whether this contributes to field splitting is unclear. Similarly, Baudouin-Gonzalez *et al*. [20] proposed that Wnt signalling restricts the eye field; we might therefore expect Wnt expression in the surrounding region to contribute to eye sizes.

Eye size is not reflected by all genes examined. *ato, dac* and *Six3.1* expression are restricted within the developing eyes and do not obviously correspond to overall size, as also observed in *C. salei* [18]. In the case of *ato* this may reflect the distribution of photoreceptors or other neural elements within the developing eyes.

### (c) Eye position

While the location of the AMEs is relatively consistent, that of the secondary eye pairs varies substantially [8]. Although the initial location of the SEP is consistent between species, the timing of its division may contribute to this diversity. In *M. muscosa* and *P. amentata*, division occurs early, around stage 10.2/11 (figures 2–4), meaning the PLEs are determined before head closure begins and therefore do not migrate anteriorly. By contrast, the division of the SEP was not reflected in *dac* expression patterns by stage 13.2 in *A. geniculata* or *P. phalangioides*, which exhibit clustered or triad arrangements. *P. tepidariorum* and *Z. x-notata* sit between these two extremes, both in terms of timing and the eventual position of the secondary eyes.

### (d) Eye identity, function and regionalization

**Figure 5.**
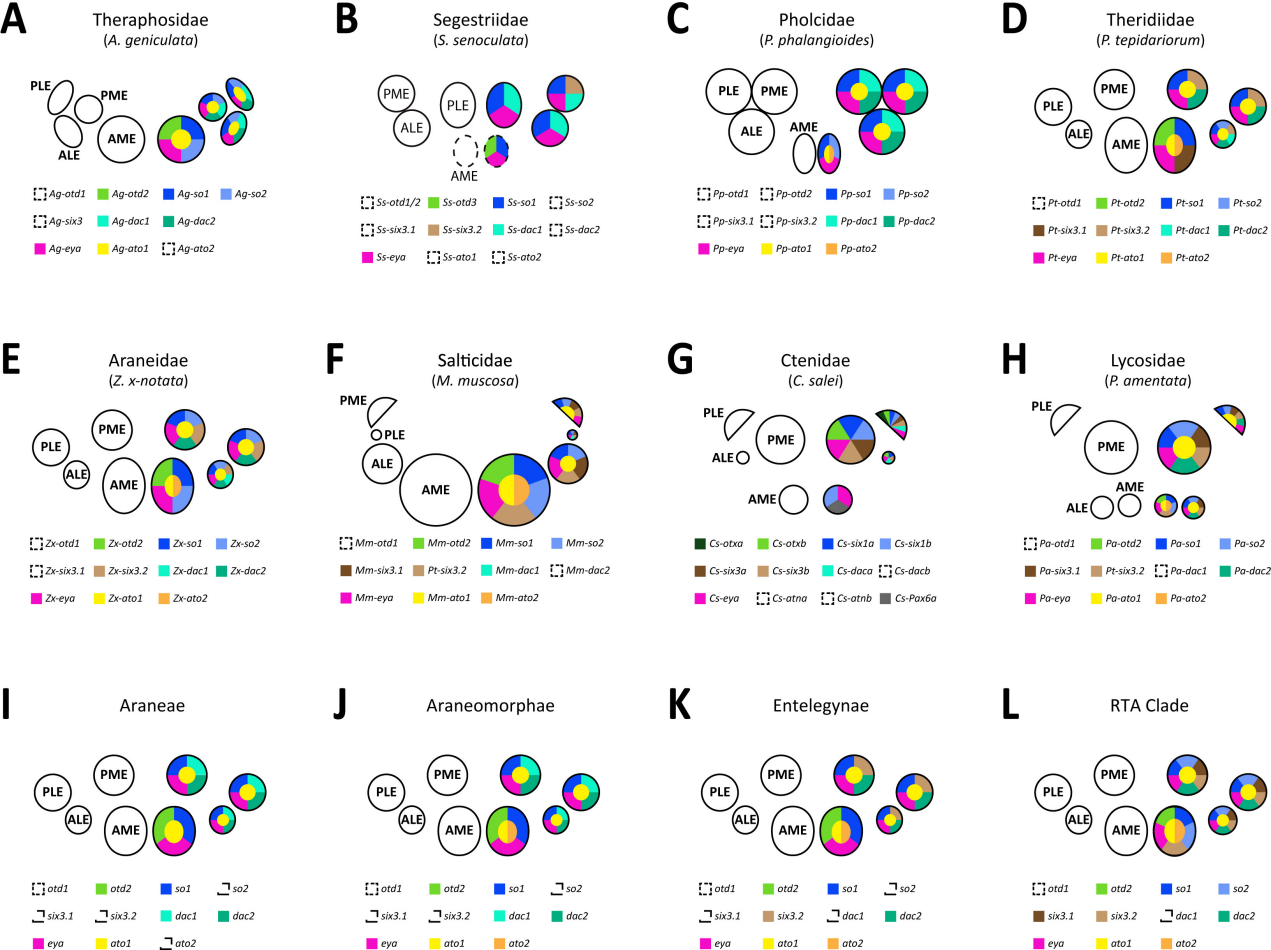
Summary of RDG expression. The combination of RDGs expressed in each eye pair demonstrates both consistency and variation across families (*A*–*H*). Phylogenetic patterns, such as the expression of *dac1* orthologues in all secondary eyes in the more plesiomorphic, non-entelegyne spiders, provide insight to likely RDG expression in the ancestors of major clades (*I–L*).

The identity of the four eye pairs may be determined by a combinatorial code of RDGs that varies between species (figure 5) [18,19]. There is particular variation among the secondary eyes, particularly in *dac* and *Six3* expression. We also report finer spatial details within or surrounding the developing eyes. *ato* expression is restricted to selected cells. In *D. melanogaster*, *ato* regulates photoreceptor differentiation [68]; Homann [55] described the differentiation of pigment and photoreceptor cells in two separate hemispheres of the retina, compatible with observed expression of *ato1*. The expression of *glass*, which acts downstream of *ato* to trigger photoreceptor differentiation in *Drosophila* [15], was also recently detected at the centre of the eyes of *P. tepidariorum* by Medina-Jiménez *et al*. [67], supporting a putative conserved role for both genes.

Whether, and how, differences in RDG expression affect eye morphology and function, remains purely speculative. However, several correlations warrant further exploration. For example, given the role of *ato* in photoreceptor differentiation, the restriction of *ato2* expression to the AMEs could be connected to the AME-specific everted, rather than inverted, photoreceptors [55]. The absence of *dac2* expression in the secondary eyes of *M. muscosa* was striking and unexpected; one unusual feature of Salticidae is that their secondary eyes lack a tapetum [55]. We also detected site-specific positive selection in *Mm-dac2*, suggesting functional change as well as differential expression. Examination of the Philodromidae, which also lack a tapetum [55], might shed further light on whether *dac2* is linked to its formation.

### (e) Eye initiation and Pax6

Our data provide no direct evidence for *Pax6* as master regulator; we did not detect unequivocal overlap between the eye primordia and *Pax6* expression in any species. However, it is plausible that the early *Pax6* expression detected by Leite *et al*. [56] indirectly triggers RDG expression in this area shortly afterwards. In the beetle *Tribolium castaneum*, *Pax6* orthologues are required to initiate the ocular segment, which later harbours precursor cells to the compound eye, larval eye and optic neuropil primordia [69]. Thus, components of the spider visual system may originate within a homologous early embryonic field of *Pax6*-expressing cells, with a similar time delay [70]. Alternatively, genes other than orthologues to insect RDGs may be responsible; Janeschik *et al*. [57] recently identified *Pax2* (orthologous to *shaven* in *Drosophila*) as a marker for the SEP. They suggested that *Pax2* might play a role in the initiation of the secondary eyes, replacing *Pax6*. The possibility of overlap between *Pax6.2* and *so* expression in *A. geniculata*, combined with positive selection in *Ag-eyg*, is particularly interesting, as these authors did not detect *Pax2* expression in this species. However, the earliest expression of *Pt-Pax2.1* was detected at stage 10.1, after *Pt-so1* activation [20].

### (f) A role for gene duplication?

Gene duplication facilitates diversification via sub- or neofunctionalization [71,72]. Large-scale events, like WGD, duplicate entire regulatory networks [23,73,74]. In spiders, this could enable divergence between the two eye types, or between pairs of secondary eyes [18,19]. However, identifying ohnologues that have functionally diverged can be challenging. Ohnologue pairs that are both expressed in the developing eyes, but with distinct expression patterns, include *so, ato, dac* and *Six3*. Of these, *Six3* expression most clearly suggests subfunctionalization: in *M. muscosa, P. amentata* and *C. salei, Six 3.1* and *Six3.2* appear to be mutually exclusive, with *Six3.2* expression surrounding *Six3.1* [18]. Clade-specific subfunctionalization between *ato1* and *ato2* is also suggested by the typical expression of the former in all eyes, but the restriction of the latter to the AMEs in araneomorphs. Greater positive selection on *ato2* than *ato1*, and on *otd1* than *otd2*, also indicate different selective regimes following duplication.

## 5. Conclusions

Spiders have exploited modularity in their visual systems to occupy a variety of ecological niches and morphospaces. We demonstrate that this is underpinned by a highly conserved RDG repertoire whose spatial and temporal expression patterns reflect aspects of morphological and functional diversity in adults. We identify candidate genes and mechanisms involved in the determination of eye size, number and position, as well as phylogenetic patterns and potential correlations to eye type and function, and evidence for a contribution of gene duplication.

## Data Availability

Synchrotron scans and transcriptome assemblies are available on Dryad [75]. Transcriptome reads are available on NCBI (BioProject PRJNA707377). Supplementary material is available online [76].
